# Health Tract, No. 279

**Published:** 1867-03

**Authors:** 


Vol. XIV. MARCH, 1867. No. 3.

HEALTH TRACT. No. 279.

### MADNESS.

"We thank Thee for the privileges of the holy Sabbath-day, for the health we enjoy, and for the proper use of our reason," were the first words of an introductory prayer, uttered by Dr. James Alexander not long before he died. It struck us at the time as one of the most comprehensive utterances we ever heard of that nature; and that the obligations to gratitude for the last item were infinite. The mind went over in an instant to the lunatic asylums which we had visited in different and distant countries.

"And for the proper use of our reason!" Without that what of worth is glorious bodily health! and vain the blessed privileges and the sweet rest of the Sabbath!

We have no recollection of another syllable that followed, of prayer or sermon; but countless times has memory recalled the words, "and the proper use of our reason;" first to the words and then to the speaker; so like a 'man of God' in all things, as they most felt he was who were nearest to him and seen him oftenest.

Madness, not hereditary, nearly always comes from some sudden shock, or from thinking too much on one thing. The former may not be provided against; the latter can be, because we are warned of its approach by an inability to sleep, which grows as the weary weeks pass on, until mind and body are hopelessly wrecked. The moment we find that troubled thoughts prevent our sleeping, a prompt and desperate effort should be made to divert the thoughts into another channel, by breaking off from all business, and in exciting adventures, or dangerous or laborous travel, wake up the nervous energies to new lines of action.

It was a dark, drizzly, cheerless November day. A furious storm was sweeping over Lake Michigan, and an old man, bareheaded, his long, lank grey hairs streaming in the wind, stood on the beach, convulsively waving a white handkerchief as if warning some one to hurry to the shore; and so he had once done in reality, when his three manly boys were out upon the waters in an open boat and he seeing the storm approach before they did, hurried to the shore to give the timely warning; they recognised him, knew what the signal meant and pulled away with all the energy of endangered men, but perished in his sight; and ever after when he saw a storm gathering he repaired to the beach, as if he still saw them, and they might be saved.

Reader! be thankful always, that no terrible calamity has never hurried you into madness.

